# Does *COMT* Play a Role in Parkinson's Disease Susceptibility across Diverse Ancestral Populations?

**DOI:** 10.1002/mds.70040

**Published:** 2025-09-23

**Authors:** Miguel Martín‐Bórnez, Nisar Shar, Mohamed Ahmed Nour, David Murphy, Inas Elsayed, Megha Shri Nanjundaswamy, Francisca Nwaokorie, Adedunni Olusanya, Nicole Kuznetsov, Sara Bandres‐Ciga, Alastair J. Noyce, Hirotaka Iwaki, Lietsel Jones, Pilar Gómez‐Garre, Pablo Mir, The Global Parkinson's Genetics Program (GP2), María Teresa Periñán

**Affiliations:** ^1^ Unidad de Trastornos del Movimiento, Servicio de Neurología, Instituto de Biomedicina de Sevilla, Hospital Universitario Virgen del Rocío/CSIC/Universidad de Sevilla Seville Spain; ^2^ Centro de Investigación Biomédica en Red sobre Enfermedades Neurodegenerativas, Instituto de Salud Carlos III Madrid Spain; ^3^ Department of clinical and Movement Neurosciences UCL Queen Square Institute of Neurology London United Kingdom; ^4^ NED University of Engineering and Technology Karachi Pakistan; ^5^ UCL Queen Square Institute of Neurology, Faculty of Brain Sciences London United Kingdom; ^6^ Faculty of Pharmacy University of Gezira Wadmedani Sudan; ^7^ Systems Biology Ireland, School of Medicine and Medical Science, University College Dublin Dublin Ireland; ^8^ Department of Neurology National Institute of Mental Health and Neurosciences India; ^9^ Department of Medical Laboratory Science, Faculty of Basic Medical Sciences, College of Medicine University of Lagos Lagos Nigeria; ^10^ Department of Pharmacology, Therapeutics and Toxicology, Faculty of Basic Medical Sciences, College of Medicine University of Lagos Lagos Nigeria; ^11^ Center for Alzheimer's and Related Dementias (CARD), National Institute on Aging and National Institute of Neurological Disorders and Stroke, National Institutes of Health Bethesda Maryland USA; ^12^ Data Tecnica Washington DC USA; ^13^ Center for Preventive Neurology, Wolfson Institute of Population Health, Faculty of Medicine and Dentistry, Queen Mary University of London London United Kingdom; ^14^ Departamento de Medicina, Facultad de Medicina Universidad de Sevilla Seville Spain

**Keywords:** cognitive impairment, COMT, dyskinesia, genetics, Parkinson's disease

## Abstract

**Background:**

The catechol‐*O*‐methyltransferase (*COMT*) gene is involved in brain catecholamine metabolism, but its association with Parkinson's disease (PD) risk remains unclear.

**Objective:**

Our aim was to investigate the relationship between *COMT* genetic variants and PD risk across diverse ancestries.

**Methods:**

We analyzed *COMT* variants in 2251 PD patients and 2835 controls of European descent using whole‐genome sequencing from the Accelerating Medicines Partnership‐Parkinson Disease (AMP‐PD), along with 20,427 PD patients and 11,837 controls from 10 ancestries using genotyping data from the Global Parkinson's Genetics Program (GP2).

**Results:**

Using the largest case–control datasets to date, no significant enrichment of *COMT* risk alleles in PD patients was observed across any ancestry group after correcting for multiple testing. Among Europeans, no correlations with cognitive decline, motor function, motor complications, or time to levodopa‐induced dyskinesia onset were observed.

**Conclusions:**

This study highlights the need for increased representation of diverse ancestries to better understand the role of *COMT* variants in PD. © 2025 The Author(s). *Movement Disorders* published by Wiley Periodicals LLC on behalf of International Parkinson and Movement Disorder Society.

Parkinson's disease (PD) is a progressive neurodegenerative disorder characterized by motor and non‐motor symptoms. Its etiology involves a complex interplay of genetic and environmental factors.^1^, ^2^

Catechol‐*O*‐methyltransferase (COMT) (OMIM: 116790) is a key enzyme in catecholamine metabolism, including dopamine and norepinephrine, and regulates dopamine levels in the prefrontal cortex by facilitating its breakdown. In PD, COMT activity influences levodopa metabolism, affecting its bioavailability and therapeutic response.^3^

Numerous studies have examined associations between *COMT* variants and PD susceptibility,^4^ particularly p.Val158Met (rs4680), and their impact on levodopa‐induced dyskinesia (LID),^5^, ^6^ cognitive decline,^7^, ^8^, ^9^ and motor fluctuations.^10^ However, findings remain inconsistent, leaving the role of *COMT* and variants in close proximity unresolved. *COMT* lies within a 1.5 Mb region commonly deleted in 22q11.2 deletion syndrome (22q11.2DS), a multisystem disorder associated with cognitive deficits, schizophrenia, epilepsy, and early‐onset PD. The clinical overlap of psychiatric and motor symptoms in 22q11.2DS supports interest in *COMT* as a candidate for neurodegeneration and PD risk.^11^

Here, we investigated whether genetic variation within *COMT* influences PD risk, LID, cognitive impairment, motor function, and complications, using array‐based genotyping and whole‐genome sequencing (WGS) data from the Global Parkinson's Genetics Program (GP2)^12^ and the Accelerating Medicines Partnership‐Parkinson Disease (AMP‐PD) initiatives.

## Methods

We analyzed AMP‐PD WGS data release 3.0 (https://amp-pd.org/), including 2251 unrelated PD patients and 2835 controls of European descent (Table S1). Additionally, we used large‐scale genotyping imputed data from GP2 release 7 (https://gp2.org/; DOI:10.5281/zenodo.10962119), comprising 20,427 PD patients and 11,837 controls across 10 ancestries: European (EUR), African Admixed (AAC), African (AFR), Ashkenazi Jews (AJ), American Admixed (AMR), Central Asian (CAS), East Asian (EAS), Middle Eastern (MDE), South Asian (SAS), and Complex Admixture History (CAH). The PD cohort included all individuals genotyped through GP2, comprising predominantly idiopathic PD cases as well as familial forms of the disease. This inclusive approach ensured that the full spectrum of disease burden was captured.

We applied the Sequence Kernel Association Test (SKAT) and its optimized version, SKAT‐O, implemented in RVTests, a software package for rare‐variant analysis, to evaluate the combined effect of *COMT* variants on PD risk.^13^ Given the limited number of rare, pathogenic coding and missense variants, we classified variants into three groups: (1) coding; (2) missense; and (3) rare (minor allele frequency [MAF] ≤3% or ≤1%).

Single nucleotide polymorphism (SNP)‐phenotype associations were tested using generalized linear models in PLINK 2.0.^14^ Logistic regressions included sex, age, and five genetic principal components (PCs) as covariates to account for population stratification. These PCs capture the major axes of genetic variation among individuals, reflecting ancestral differences within the study population.^15^ Bonferroni correction was applied to adjust *P*‐values for all *COMT* variants within each ancestry independently.

Clinical data from participants of European ancestry in GP2 and AMP‐PD were used to assess the impact of *COMT* variants on cognitive and motor function and the development of LID. Cognitive decline was assessed using the Montreal Cognitive Assessment (MoCA) scores (GP2: n = 568; AMP‐PD: n = 1341) with linear regressions adjusted for age, sex, education, and PCs. Associations with Movement Disorders Society (MDS) Unified Parkinson's Disease Rating Scale (UPDRS) parts III (GP2: n = 1268; AMP‐PD: n = 1666) and IV (GP2: n = 359; AMP‐PD: n = 1794) were also evaluated. LID (GP2: n = 463) was analyzed using Cox proportional hazards models with time‐to‐LID onset adjusted for age, sex, PCs, and levodopa equivalent daily dose (LEDD). Kaplan–Meier survival curves were used to visualize time‐to‐LID by *COMT* variant status.

## Results

Leveraging AMP‐PD WGS data, we identified 491 *COMT* variants: 444 intronic, 13 exonic (8 synonymous, 4 missense, 1 nonframeshift [3‐bp] deletion), and 34 UTR variants (Table S2). None were significantly associated with PD risk (Table S3).

**FIG. 1:**
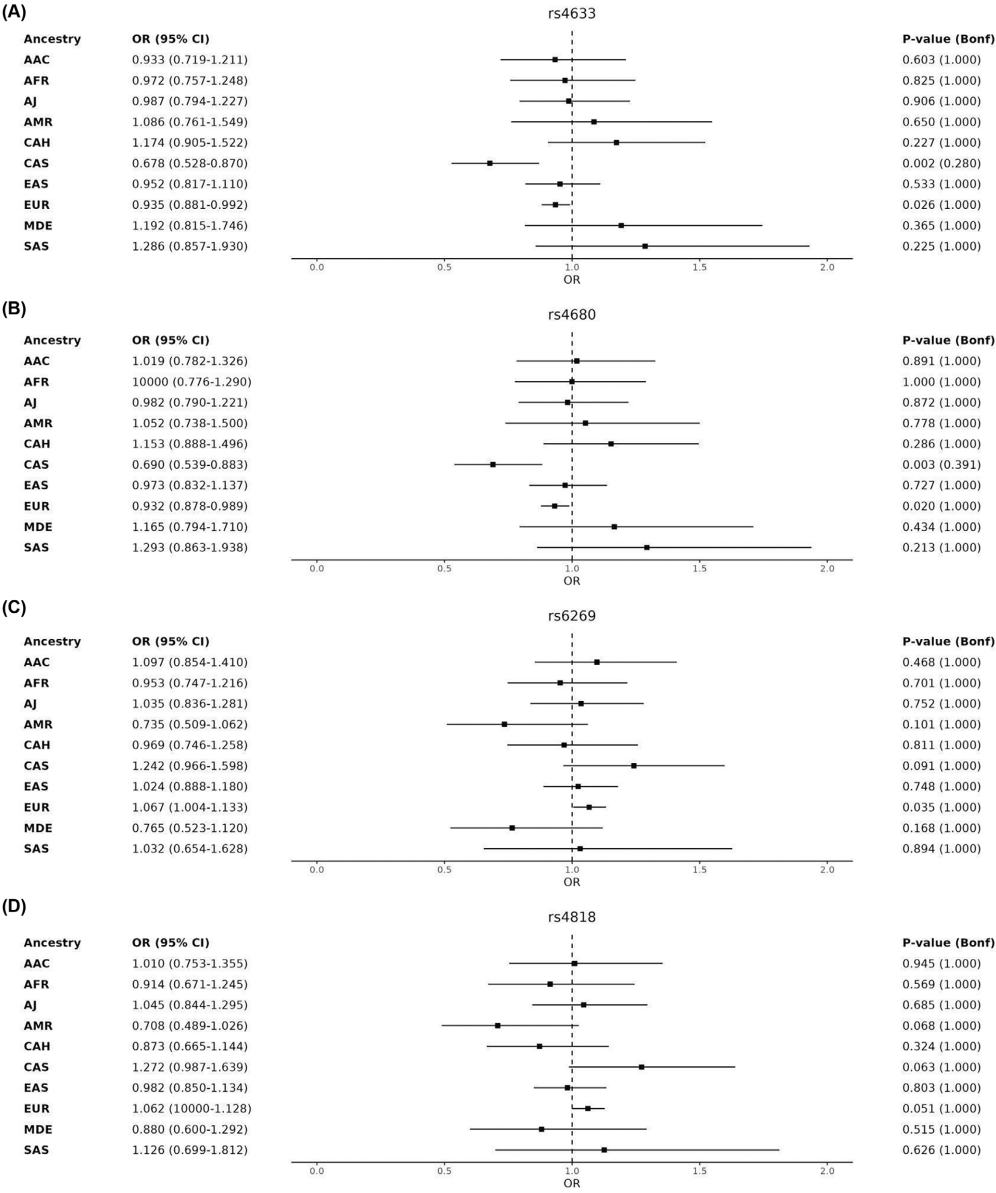
Forest plot illustrating the association between Parkinson's disease risk and the four most extensively studied *COMT* variants (rs4633, rs4680, rs6269, rs4818) across 10 ancestries. The analysis was performed using a generalized linear model adjusted for age, sex, and five principal components. (A) rs4633, (B) rs4680, (C) rs6269, and (D) rs4818. AAC, African Admixed; AFR, African; AJ, Ashkenazi Jews; AMR, American Admixed; Bonf, Bonferroni; CAS, Central Asian; CI, confidence intervals; EAS, East Asian; EUR, European; MDE, Middle Eastern; OR, odds ratio; SAS, South Asian.

For the GP2 genotyping data, all identified variants are listed in Table S2. Nominal associations with PD risk were observed across multiple ancestries (Table 1). In CAS, p.Val158Met (rs4680) and p.His62 = (rs4633) were associated with reduced PD risk (odds ratio [OR], ~0.70). In EUR, the same variants showed nominal associations with smaller effects, whereas c.‐98A>G (rs6269) was linked to increased PD risk. In AJ and MDE, *c.764C>T (rs165728) showed opposing effects. In EAS, p.Ala72Ser (rs6267) had nominal association with PD risk. However, none remained significant after multiple testing correction (Table S4). Recent PD genome‐wide association study (GWAS) meta‐analyses^16^, ^17^, ^18^, ^19^, ^20^ found no GWAS‐significant *COMT* variant associations (*P* < 5 × 10^−8^) (Fig. S1). Figure 1 illustrates ancestry‐specific associations for four key *COMT* variants (rs4680, rs4633, rs6269, and rs4818), highlighting variations in effect size and direction across populations.

**Table 1:** *COMT* variants showing nominal associations with PD in the GP2 genotyping dataset

Variant	GP2 ancestry	Hom cases	Het cases	Total cases	Carrier freq in cases	Hom controls	Het controls	Total controls	Carrier freq in controls	A1	OR (95% CI)	*P* (BONF)
chr22:19963748:G:A (rs4680; p.Val158Met)	CAS	72	248	519	0.378	76	135	306	0.472	A	0.690 (0.539–0.883)	0.003 (0.391)
	EUR	3145	6356	13,034	0.500	1331	2502	5191	0.513	A	0.932 (0.878–0.989)	0.020 (1)
chr22:19962712:C:T (rs4633; p.His62=)	CAS	71	243	519	0.377	75	135	306	0.473	T	0.678 (0.528–0.87)	0.002 (0.280)
	EUR	3152	6312	13,034	0.484	1198	2506	5191	0.472	T	0.935 (0.881–0.992)	0.026 (1)
chr22:19962429:A:G (rs6269; c.‐98A>G)	EUR	2221	6247	13,034	0.416	807	2516	5191	0.404	G	1.067 (1.004–1.133)	0.035 (1)
chr22:19969500:C:T (rs165728; c.*764C>T)	AJ	1	93	1234	0.038	2	45	391	0.063	C	0.602 (0.373–0.971)	0.038 (1)
	MDE	2	21	230	0.055	0	13	196	0.033	C	2.475 (1.030–5.949)	0.043 (1)
chr22:19962740:G:T (rs6267; p.Ala72Ser)	EAS	1	118	2547	0.025	0	91	2345	0.021	T	0.643 (0.415–0.998)	0.049 (1)

Results from logistic regression models adjusted for age, sex, and five principal components to account for population stratification.

Abbreviations: PD, Parkinson's disease; GP2, The Global Parkinson's Genetics Program; Hom, homozygous; Het, heterozygous; OR, odds ratio; CI, confidence intervals; BONF, Bonferroni; CAS, Central Asian; EUR, European; AJ, Ashkenazi Jews; MDE, Middle Eastern; EAS, East Asian.

Gene‐based burden analysis showed nominal associations with PD risk for coding (n = 33; SKAT *P* = 0.023, SKAT‐O *P* = 0.022) and missense variants (n = 18; SKAT *P* = 0.022, SKAT‐O *P* = 0.019) in the GP2 EUR group. Missense variants in AJ were also nominally associated with PD (n = 3; SKAT‐O *P* = 0.036). Rare variants with MAF <1% in the AFR group showed nominal association with PD risk (n = 232; SKAT‐O *P* = 0.028). In AMP‐PD, coding variants showed nominal association (n = 12; SKAT‐O *P* = 0.010). However, none remained significant after multiple testing correction and should be therefore interpreted with caution (Table S5).

Given their potential clinical relevance, we examined associations between *COMT* variants and cognitive as well as motor outcomes. Both p.Val158Met (A1 = G; ꞵ = −0.338; standard error [SE] = 0.150, *P* = 0.024, Bonferroni‐corrected *P* = 1) and p.His62 = (A1 = C; ꞵ = −0.330; SE = 0.149, *P* = 0.027, Bonferroni‐corrected *P* = 1) showed nominal associations with MoCA in GP2 EUR. Similarly, these variants were nominally associated with MDS‐UPDRS part IV scores in AMP‐PD, along with c.*764C>T (A1 = T; ꞵ = 0.666; SE = 0.320, *P* = 0.038, Bonferroni‐corrected *P* = 1). However, none remained significant after multiple testing (Tables S6–S9).

In the 463 EUR PD patients from GP2, 34.3% developed LID. The analysis focused on four *COMT* variants (rs6269, rs4633, rs4818, and rs4680) selected for their functional significance in modulating COMT activity.^21^ No significant associations with time‐to‐LID onset were found using Cox proportional hazards model (*P* > 0.05). Kaplan–Meier survival curves are presented in Figure S2.

## Discussion

This study leveraged AMP‐PD WGS and GP2 genotyping data, representing the largest and most ancestrally diverse dataset to date, to analyze *COMT* variants and their impact on PD across populations. Recent work by Poplawska et al^22^ emphasizes the lack of ethnic diversity in COMT inhibitor trials, underscoring the importance of including underrepresented populations in genetic studies of *COMT* and PD.

Despite this broad ancestry coverage, no statistically significant associations between *COMT* variants and PD risk were observed in any group, including EAS, SAS, and EUR, which have been the focus of prior research.^4^, ^9^, ^23^, ^24^ Significant associations have been reported in Asians, particularly among Japanese and Chinese cohorts (EAS), and to a lesser extent in Indian cohorts (SAS).^23^, ^24^, ^25^ For instance, a study of 109 Japanese PD patients and 153 controls found a significant association with the homozygous p.Val158Met genotype,^26^ supported by a meta‐analysis including 1581 PD patients and 1376 controls from EAS (Japan and China), where significance was limited to the Japanese subgroup.^25^ Another meta‐analysis by Wang et al^24^ similarly found an effect only in the Japanese subgroup when stratified by ethnicity. These findings suggest that effects within the broader EAS population may be driven by specific subcohorts. Therefore, adjusting for population stratification using PCs, as done in our analysis, is essential. Despite >80% power to detect an OR ≥1.5 for variants with MAF ≥10%, our large EAS cohort (2646 cases; 2453 controls) did not replicate earlier associations.

In SAS populations, Wang et al^24^ also reported a significant association between p.Val158Met and PD risk in 489 Indian PD patients and 823 controls, which we did not replicate. Although our study is among the largest to date, the relatively small SAS subgroup (319 cases; 198 controls) may lack power to detect modest effect sizes. Future GP2 data releases with larger sample sizes from underrepresented populations will improve robustness and generalizability of findings.

Our research represents the most comprehensive study of p.Val158Met in Europeans. To date, no well‐powered study has demonstrated a significant association in this group. Meta‐analyses including up to 11,428 PD cases and 16,726 controls have consistently reported no significant link between this variant and PD susceptibility in Europeans.^4^, ^9^ Our findings align with these results and further reinforce them by leveraging harmonized, ancestry‐stratified data with robust statistical power. Moreover, multiple large GWAS have not identified p.Val158Met or other *COMT* variants as genome‐wide significant loci for PD.^17^, ^19^ Collectively, the current evidence strongly suggests that *COMT* is unlikely to play a role on PD risk in Europeans.

LID is a major complication of long‐term levodopa therapy, affecting 20% to 40% of PD patients.^27^ In our dataset, 34.3% of patients developed LID, all on levodopa treatment. We observed no significant associations between *COMT* variants and LID in Europeans, consistent with a meta‐analysis reporting no genome‐wide significant *COMT* associations with LID.^27^ Nonetheless, some studies have suggested that rs4680 may influence LID risk in PD patients of European,^28^ Asian, and Brazilian ancestry.^5^, ^6^, ^10^

We also examined motor function and complications using MDS‐UPDRS parts III and IV in the GP2 EUR group. No significant associations were observed after multiple testing correction. This aligns with a meta‐analysis of 1574 PD patients, stratified by Asian and Caucasian populations, reporting a significant association between rs4680 and higher UPDRS part III scores only in the Asian subgroup (n = 393).^9^

Consistent with a previous meta‐analysis, we found no significant association between *COMT* variants and cognitive decline within the GP2 EUR group.^9^ However, given the role of *COMT* in modulating dopamine availability in the prefrontal cortex, a key region for executive function, we cannot rule out a potential influence on cognition.^29^, ^30^ Only 2.5% of our patients scored <21 on the MoCA, indicating low prevalence of dementia that may partly explain the lack of significant associations.

In conclusion, our results show no association between *COMT* and PD risk that passes multiple test corrections. These findings highlight the importance of increased representation of diverse ancestries to better understand the role of *COMT* variants in PD.

## Author Roles

(1) Research Project: A. Conception, B. Organization, C. Execution; (2) Statistical Analysis: A. Design, B. Execution, C. Review and Critique; (3) Manuscript Preparation: A. Writing of the First Draft, B. Review and Critique.

M.M.‐B.: 1B, 1C, 2B, 3A.

N.S.: 1C, 3A.

M.A.N.: 1C, 3A.

D.M.: 1C, 3A.

I.E.: 1C, 3A.

M.S.N.: 1C, 3A.

F.N.: 1C, 3A.

A.O.: 1C, 3A.

N.K.: 1C, 2C, 3B.

S.B.C.: 2C, 3B.

A.J.N.: 2C, 3B.

H.I.: 1B, 2B.

L.J.: 1B, 2B.

P.G.‐G.: 2C, 3B.

P.M.: 2C, 3B.

M.T.P.: 1A, 1B, 2A, 3A, 3B.

## Financial Disclosures

This research was supported in part by the Intramural Research Program of the National Institutes of Health, National Institute on Aging, National Institutes of Health, Department of Health and Human Services; project number ZO1 AG000535 and ZIA AG000949, as well as the National Institute of Neurological Disorders and Stroke (NINDS) and the National Human Genome Research Institute. This project was supported by the GP2 (https://gp2.org). GP2 is funded by the ASAP initiative and implemented by The Michael J. Fox Foundation for Parkinson's Research (MJFF). For a complete list of GP2 members see doi.org/10.5281/zenodo.7904831. The AMP‐PD program is a public–private partnership managed by the Foundation for the National Institutes of Health and funded by the NINDS in partnership with the ASAP initiative; Celgene Corporation, a subsidiary of Bristol‐Myers Squibb Company; GlaxoSmithKline PLC (GSK); MJFF; Pfizer; Sanofi US Services; and Verily Life Sciences. M.M.B. is supported by a predoctoral contract for training in health research from the Instituto de Salud Carlos III (FI22/00226). M.S.N. received funding support from CSIR under sanction no. 09/0490(16118)/2022‐EMR‐I and the Parkinson's Disease and Movement Disorders Research Fund (file no. 13020).

## Supporting information


**Table S1.** Summary statistics of the GP2 genotyping and AMP‐PD WGS.
**Table S2.** *COMT* variants identified in AMP‐PD WGS and GP2 genotyping data.
**Table S3.** *COMT exonic* variants associated with PD risk adjusted by age at baseline, sex, and five principal components in AMP‐PD WGS data.
**Table S4.** *COMT exonic* variants associated with PD risk adjusted by age at baseline, sex, and five principal components in GP2 genotyping data.
**Table S5.** Gene burden analysis of *COMT* variants. N Var, number of variants; AAC: African Admixed; AFR: African; AJ: Ashkenazi Jews; AMR: American Admixed; CAH, Complex Admixture History; CAS: Central Asian; EAS: East Asian; EUR: European; MDE: Middle Eastern; SAS: South Asian.
**Table S6.** *COMT* exonic variants associated with MoCA scores adjusted by age at baseline, sex, education level, and five principal components in GP2 European genotyping data (N = 568).
**Table S7.** *COMT* exonic variants associated with MOCA scores adjusted by age at baseline, sex, education level, and five principal components in AMP‐PD WGS (N = 1341). A1: Allele 1, effect allele; BONF, Bonferroni adjusted *P*‐value.
**Table S8.** *COMT* exonic variants associated with MDS UPDRS Parts III (N = 1268) and IV (N = 359) total scores, adjusted by age at baseline, sex, education level, and five principal components in GP2 European genotyping data.
**Table S9.** *COMT* exonic variants associated with MDS UPDRS III (N = 1666) and IV (N = 1794) scores adjusted by age at baseline, sex, education level and five principal components in AMP‐PD WGS.
**Figure S1.** Locus zoom plot for *COMT* variants versus PD risk. (A) Data was obtained from the latest European PD GWAS meta‐analysis excluding 23andMe data, consisting of 15056 PD cases, 18,618 UK Biobank proxy‐cases, and 449,056 healthy controls (1). (B) Data was obtained from the East Asian PD GWAS, consisting of 6724 PD cases and 24,851 healthy controls (2). (C) Data was obtained from the Latin American PD GWAS, consisting of 807 PD cases and 690 healthy controls (3). (D) Data was obtained from the African PD GWAS excluding 23andMe data, consisting of 1200 PD cases and 2445 healthy controls (4). (E) Data was obtained from the multi‐ancestry PD GWAS excluding 23andMe data, consisting of 25,374 PD cases, 18,618 proxy‐cases, and 571,138 healthy controls (5). *P*‐values on the log‐10 scale are plotted along the horizontal axis with the gene names and size of the flanking region. The most strongly associated SNPs are indicated by a purple diamond and pairwise LD (r2) with these SNPs are indicated by dotted color as described in the legend in the upper right corner. The right vertical axis indicates the regional recombination rate (cM/Mb) which is overlaid in blue. LD, linkage disequilibrium; PD, Parkinson's disease; SNP, single nucleotide polymorphism.
**Figure S2.** Levodopa‐induced Dyskinesias (LID) survival analysis among 463 PD patients from GP2 European genotyping data.

## Data Availability

Data used in the preparation of this article were obtained from GP2 (https://gp2.org). Specifically, we used Tier 2 data from GP2 release 7 (DOI:10.5281/zenodo.10962119). Tier 1 data can be accessed by completing a form on the AMP‐PD website (https://amp-pd.org/register-for-amp-pd). Tier 2 data access requires approval and a Data Use Agreement signed by your institution. All code generated for this article, and the identifiers for all software programs and packages used, are available on GitHub (https://github.com/GP2code/COMT-PD-GeneAnalysis) and were given a persistent identifier via Zenodo (DOI 10.5281/zenodo.15185052).
