# Supplementary material for: Does COMT Play a Role in Parkinson's Disease Susceptibility across Diverse Ancestral Populations?
Source: Mov Disord. 2025 Sep 23;40(12):2819–24. doi: 10.1002/mds.70040 (PMC12710204; doi:10.1002/mds.70040)
Supplement: Supplementary file 1 — Table S1. Summary statistics of the GP2 genotyping and AMP‐PD WGS. Table S2. COMT variants identified in AMP‐PD WGS and GP2 genotyping data. Table S3. COMT exonic variants associated with PD risk adjusted by age at baseline, sex, and five principal components in AMP‐PD WGS data. Table S4. COMT exonic variants associated with PD risk adjusted by age at baseline, sex, and five principal components in GP2 genotyping data. Table S5. Gene burden analysis of COMT variants. N Var, number of variants; AAC: African Admixed; AFR: African; AJ: Ashkenazi Jews; AMR: American Admixed; CAH, Complex Admixture History; CAS: Central Asian; EAS: East Asian; EUR: European; MDE: Middle Eastern; SAS: South Asian. Table S6. COMT exonic variants associated with MoCA scores adjusted by age at baseline, sex, education level, and five principal components in GP2 European genotyping data (N = 568). Table S7. COMT exonic variants associated with MOCA scores adjusted by age at baseline, sex, education level, and five principal components in AMP‐PD WGS (N = 1341). A1: Allele 1, effect allele; BONF, Bonferroni adjusted P‐value. Table S8. COMT exonic variants associated with MDS UPDRS Parts III (N = 1268) and IV (N = 359) total scores, adjusted by age at baseline, sex, education level, and five principal components in GP2 European genotyping data. Table S9. COMT exonic variants associated with MDS UPDRS III (N = 1666) and IV (N = 1794) scores adjusted by age at baseline, sex, education level and five principal components in AMP‐PD WGS. Figure S1. Locus zoom plot for COMT variants versus PD risk. (A) Data was obtained from the latest European PD GWAS meta‐analysis excluding 23andMe data, consisting of 15056 PD cases, 18,618 UK Biobank proxy‐cases, and 449,056 healthy controls (1). (B) Data was obtained from the East Asian PD GWAS, consisting of 6724 PD cases and 24,851 healthy controls (2). (C) Data was obtained from the Latin American PD GWAS, consisting of 807 PD cases and 690 healthy controls (3) [file MDS-40-2819-s001.docx]

**Supplementary Material**

**Supplementary Methods**

We analyzed AMP-PD WGS data release 3.0 (<https://amp-pd.org/>), including 2,251 unrelated PD patients and 2,835 controls of European descent (Table S1). Additionally, we utilized large-scale genotyping imputed data from GP2 release 7 (<https://gp2.org/>; DOI:10.5281/zenodo.10962119), comprising 20,427 PD patients and 11,837 controls across ten ancestry populations: European (EUR), African Admixed (AAC), African (AFR), Ashkenazi Jews (AJ), American Admixed (AMR), Central Asian (CAS), East Asian (EAS), Middle Eastern (MDE), South Asian (SAS), and Complex Admixture History (CAH). The PD cohort included all individuals genotyped through GP2, comprising predominantly idiopathic PD cases as well as familial forms of the disease. This inclusive approach ensured that the full spectrum of disease burden was captured.

The GenoTools pipeline (<https://github.com/GP2code/GenoTools>) was used for genetic ancestry prediction by calculating genetic principal components (PCs), and quality control (QC) on genotyping data was conducted according to previously described methods [1]. Variants with a Hardy-Weinberg Equilibrium (HWE) p-value$\leq$1x10^-4^ in control samples were removed after preliminary QC. Variants were pruned to exclude those with a minor allele frequency (MAF)$\leq$1% and a minor allele count of 2. *COMT* gene positions were obtained from Ensembl (<https://www.ensembl.org>). Variants were annotated using ANNOVAR [[31]](https://paperpile.com/c/zs6UCi/vs9PQ). We applied the Sequence Kernel Association Test (SKAT) and its optimized version, SKAT-O, using RVTESTS to evaluate the combined effect of multiple *COMT* variants on PD risk [13]. Given the limited number of rare, pathogenic coding and missense variants, we classified variants into three analysis groups: (i) coding variants, (ii) missense variants, and (iii) rare variants (MAF≤3% or ≤1%).

SNP-phenotype associations were tested using a generalized linear model in PLINK 2.0 [[14]](https://paperpile.com/c/zs6UCi/N0PRU). Logistic regression models included sex, age, and the first five genetic principal components (PCs) as covariates to control for population stratification. Bonferroni correction was applied to adjust p-values for all *COMT* variants within each ancestry independently. Power calculations were performed using the online Genetic Association Study (GAS) Power Calculator (<https://csg.sph.umich.edu/abecasis/cats/gas_power_calculator/index.html>).

We examined clinical data from participants of European ancestry in GP2 and AMP-PD to assess the impact of *COMT* variants on cognitive and motor function and the development of LID. Cognitive decline was assessed using the Montreal Cognitive Assessment (MoCA) scores (GP2: N=568; AMP-PD N:=1,341) with linear regression models, adjusting for age, sex, education, and PCs. Associations with MDS-UPDRS Parts III (GP2: N=1,268; AMP-PD N:=1,666) and Part IV scores (GP2: N=359; AMP-PD: N=1,794) were also evaluated. LID (GP2: N=463) associations were analyzed using Cox proportional hazards models, incorporating time-to-LID onset data and adjusting for age, sex, PCs, and levodopa equivalent daily dose (LEDD). Kaplan-Meier survival curves were used to visualize time-to-LID onset by *COMT* variant status.

**Table S1.** Summary statistics of the GP2 genotyping and AMP-PD WGS.

*Abbreviations: AAC, African Admixed; AFR, African;* AJ, Ashkenazi Jews; *AMR, American Admixed; CAS, Central Asian; SD, Standard deviation; EAS, East Asian; EUR, European; MDE, Middle Eastern; SAS, South Asian; CAH, Complex Admixture History; WGS, Whole Genome Sequencing.*

| **Ancestry** | **Cohort** | ***N* Total**  ***(n* PD*, n* controls*)*** | **Age, *y* (Mean ± SD)** | **Male sex, *n* (%)** |
| --- | --- | --- | --- | --- |
| AAC | GP2 | 1072 (279, 793) | 65.83 ± 10.37 | 437 (40.8) |
| AFR | GP2 | 2546 (929, 1617) | 63.48 ± 14.92 | 1432 (56.2) |
| AJ | GP2 | 1625 (1234, 391) | 69.76 ± 9.85 | 1097 (67.5) |
| AMR | GP2 | 577 (427,150) | 61.83 ± 11.95 | 327 (56.7) |
| CAH | GP2 | 777 (494, 283) | 55.28 ± 17.42 | 409 (52.6) |
| CAS | GP2 | 825 (519, 306) | 59.03 ± 9.64 | 386 (46.8) |
| EAS | GP2 | 4892 (2547, 2345) | 64.96 ± 10.96 | 3105 (63.5) |
| SAS | GP2 | 517 (319, 198) | 59.50 ± 15.62 | 336 (65.0) |
| MDE | GP2 | 426 (230, 196) | 60.12 ± 11.28 | 267 (62.7) |
| EUR | GP2 | 18225 (13034, 5191) | 66.79 ± 11.21 | 11180 (61.3) |
|  | AMP-PD | 5086 (2251, 2835) | 66.91 ± 11.84 | 2803 (55.1) |

**Table S2.** *COMT* variants identified in AMP-PD WGS and GP2 genotyping data.

*Abbreviations: AAC, African Admixed; AFR, African;* AJ, Ashkenazi Jews; *AMR, American Admixed; CAS, Central Asian; CI, confidence intervals; EAS, East Asian; EUR, European; MDE, Middle Eastern; SAS, South Asian; CAH, Complex Admixture History; WGS, Whole Genome Sequencing.*

| **Cohort** | **Ancestry** | **Total Variants** | **Intronic** | **3’-UTR** | **5’-UTR** | **Coding** | | |
| --- | --- | --- | --- | --- | --- | --- | --- | --- |
|  |  |  |  |  |  | **Synonymous** | **Nonsynonymous** | **Nonframeshift deletion** |
| AMP-PD | EUR | 491 | 444 | 20 | 14 | 8 | 4 | 1 |
| GP2 | EUR | 971 | 865 | 39 | 32 | 15 | 18 | 2 |
|  | AFR | 476 | 432 | 21 | 9 | 6 | 8 | 0 |
|  | EAS | 412 | 366 | 15 | 12 | 11 | 8 | 0 |
|  | AMR | 245 | 219 | 12 | 5 | 7 | 2 | 0 |
|  | CAS | 224 | 207 | 6 | 4 | 5 | 2 | 0 |
|  | AJ | 242 | 214 | 12 | 6 | 7 | 3 | 0 |
|  | SAS | 200 | 180 | 8 | 3 | 5 | 4 | 0 |
|  | MDE | 200 | 181 | 8 | 3 | 6 | 2 | 0 |
|  | AAC | 406 | 372 | 14 | 11 | 5 | 4 | 0 |
|  | CAH | 357 | 324 | 15 | 8 | 6 | 4 | 0 |

**Table S3.** *COMT exonic* variants associated with PD risk adjusted by age at baseline, sex, and five principal components in AMP-PD WGS data.

*Abbreviations: A1, Allele 1, effect allele; OR, odds ratio; CI, confidence intervals; BONF, Bonferroni p-value correction.*

| **Variant** | **Hom Cases** | **Het Cases** | **Total Cases** | **Carrier freq in Cases** | **Hom Controls** | **Het Controls** | **Total Controls** | **Carrier freq in Controls** | **A1** | **OR (95% CI)** | ***p* (BONF)** |
| --- | --- | --- | --- | --- | --- | --- | --- | --- | --- | --- | --- |
| chr22:19962429:A:G  (rs6269; c.-98A>G) | 415 | 1081 | 2251 | 0.664594 | 468 | 1377 | 2835 | 0.650794 | G | 1.098 (1.008 - 1.195) | 0.032 (1) |
| chr22:19962712:C:T  (rs4633; p.His62=) | 553 | 1116 | 2251 | 0.494 | 746 | 1424 | 2835 | 0.514 | T | 0.905 (0.832 - 0.984) | 0.019 (1) |
| chr22:19963684:C:G  (rs4818; p.Leu136=) | 411 | 1072 | 2251 | 0.421 | 453 | 1362 | 2835 | 0.400 | G | 1.109 (1.018 - 1.207) | 0.018 (1) |
| chr22:19963748:G:A  (rs4680; p.Val158Met) | 552 | 1114 | 2251 | 0.493 | 739 | 1426 | 2835 | 0.512 | A | 0.909 (0.836 - 0.989) | 0.027 (1) |
| chr22:19964281:G:A  (rs769224; p.Pro199=) | 1 | 65 | 2251 | 0.015 | 1 | 116 | 2835 | 0.021 | A | 0.718 (0.519 - 0.994) | 0.046 (1) |
| chr22:19964293:C:T  (rs165631; p.Leu203=) | 0 | 47 | 2251 | 0.010 | 3 | 101 | 2835 | 0.019 | T | 0.738 (0.508 - 1.071) | 0.109 (1) |
| chr22:19969030:C:G  (rs9332381; c.*294C>G) | 2 | 138 | 2251 | 0.032 | 4 | 205 | 2835 | 0.038 | G | 0.843 (0.667 - 1.066) | 0.153 (1) |
| chr22:19969258:G:A  (rs165599; c.*522G>A) | 194 | 941 | 2251 | 0.50422 | 261 | 1188 | 2835 | 0.511111 | A | 0.999 (0.912 - 1.096) | 0.991 (1) |
| chr22:19969340:G:A  (rs36082074; c.*604G>A) | 2 | 136 | 2251 | 0.031 | 3 | 159 | 2835 | 0.029 | A | 1.221 (0.954 - 1.563) | 0.113 (1) |
| chr22:19969362:T:C  (rs35478083; c.*626T>C) | 2 | 133 | 2251 | 0.030 | 3 | 201 | 2835 | 0.037 | C | 0.832 (0.656 - 1.055) | 0.130 (1) |
| chr22:19969500:C:T  (rs165728; c.*764C>T) | 5 | 213 | 2251 | 0.050 | 7 | 249 | 2835 | 0.046 | C | 0.995 (0.818 - 1.208) | 0.956 (1) |

**Table S4.** *COMT exonic* variants associated with PD risk adjusted by age at baseline, sex, and five principal components in GP2 genotyping data.

*Abbreviations: A1, Allele 1, effect allele; OR, odds ratio; CI, confidence intervals; BONF, Bonferroni p-value correction; AAC: African Admixed; AFR: African; AJ: Ashkenazi Jews; AMR: American Admixed; CAS: Central Asian; EAS: East Asian; EUR: European; MDE: Middle Eastern; SAS: South Asian.*

| **GP2 Ancestry** | **Variant** | **Hom Cases** | **Het Cases** | **Total Cases** | **Carrier freq in Cases** | **Hom Controls** | **Het Controls** | **Total Controls** | **Carrier freq in Controls** | **A1** | **OR (95% CI)** | ***p* (BONF)** |
| --- | --- | --- | --- | --- | --- | --- | --- | --- | --- | --- | --- | --- |
| EUR | chr22:19962429:A:G  (rs6269; c.-98A>G) | 2221 | 6247 | 13034 | 0.416 | 807 | 2516 | 5191 | 0.404 | G | 1.067 (1.004-1.133) | 0.035 (1) |
| AFR |  | 135 | 392 | 929 | 0.357 | 230 | 747 | 1617 | 0.374 | G | 0.953 (0.747-1.216) | 0.700 (1) |
| EAS |  | 280 | 1110 | 2547 | 0.328 | 224 | 1042 | 2345 | 0.318 | G | 1.024 (0.888-1.18) | 0.748 (1) |
| AAC |  | 43 | 137 | 279 | 0.400 | 110 | 373 | 793 | 0.374 | G | 1.097 (0.854-1.410) | 0.468 (1) |
| AJ |  | 279 | 604 | 1234 | 0.471 | 87 | 194 | 391 | 0.471 | G | 1.035 (0.836-1.281) | 0.752 (1) |
| AMR |  | 48 | 176 | 427 | 0.319 | 20 | 76 | 150 | 0.387 | G | 0.735 (0.509-1.062) | 0.101 (1) |
| SAS |  | 38 | 128 | 319 | 0.320 | 18 | 88 | 198 | 0.313 | G | 1.032 (0.654-1.628) | 0.894 (1) |
| CAH |  | 66 | 224 | 494 | 0.361 | 33 | 142 | 283 | 0.369 | G | 0.969 (0.746-1.258) | 0.811 (1) |
| CAS |  | 77 | 269 | 519 | 0.408 | 48 | 128 | 306 | 0.366 | G | 1.242 (0.966-1.598) | 0.091 (1) |
| MDE |  | 43 | 100 | 230 | 0.404 | 57 | 87 | 196 | 0.513 | G | 0.765 (0.523-1.12) | 0.168 (1) |
| EUR | chr22:19962712:C:T  (rs4633; p.His62=) | 3152 | 6312 | 13034 | 0.484 | 1198 | 2506 | 5191 | 0.472 | T | 0.935 (0.881-0.992) | 0.026 (1) |
| AFR |  | 78 | 341 | 929 | 0.271 | 153 | 670 | 1617 | 0.306 | T | 0.972 (0.757-1.248) | 0.825 (1) |
| EAS |  | 155 | 795 | 2547 | 0.249 | 141 | 797 | 2345 | 0.266 | T | 0.952 (0.817-1.11) | 0.533 (1) |
| AAC |  | 23 | 140 | 279 | 0.333 | 96 | 359 | 793 | 0.347 | T | 0.933 (0.719-1.211) | 0.603 (1) |
| AJ |  | 263 | 625 | 1234 | 0.466 | 84 | 192 | 391 | 0.462 | T | 0.987 (0.794-1.227) | 0.906 (1) |
| AMR |  | 68 | 219 | 427 | 0.419 | 23 | 79 | 150 | 0.419 | T | 1.086 (0.761-1.549) | 0.65 (1) |
| SAS |  | 65 | 145 | 319 | 0.431 | 38 | 104 | 198 | 0.457 | T | 1.286 (0.857-1.93) | 0.225 (1) |
| CAH |  | 82 | 250 | 494 | 0.419 | 40 | 136 | 283 | 0.382 | T | 1.174 (0.905-1.522) | 0.227 (1) |
| CAS |  | 71 | 243 | 519 | 0.377 | 75 | 135 | 306 | 0.473 | T | 0.678 (0.528-0.87) | 0.002 (0.280) |
| MDE |  | 56 | 109 | 230 | 0.483 | 41 | 83 | 196 | 0.425 | T | 1.193 (0.815-1.746) | 0.365 (1) |
| EUR | chr22:19963684:C:G  (rs4818; p.Leu136=) | 2164 | 6224 | 13034 | 0.410 | 787 | 2499 | 5191 | 0.399 | G | 1.062 (1-1.128) | 0.051 (1) |
| AFR |  | 23 | 252 | 929 | 0.162 | 56 | 465 | 1617 | 0.180 | G | 0.914 (0.671-1.245) | 0.569 (1) |
| EAS |  | 268 | 1084 | 2547 | 0.325 | 206 | 1030 | 2345 | 0.314 | G | 0.982 (0.85-1.134) | 0.803 (1) |
| AAC |  | 12 | 108 | 279 | 0.238 | 39 | 262 | 793 | 0.215 | G | 1.010 (0.753-1.355) | 0.945 (1) |
| AJ |  | 273 | 610 | 1234 | 0.468 | 85 | 195 | 391 | 0.467 | G | 1.045 (0.844-1.295) | 0.685 (1) |
| AMR |  | 42 | 172 | 427 | 0.300 | 20 | 73 | 150 | 0.377 | G | 0.708 (0.489-1.026) | 0.068 (1) |
| SAS |  | 31 | 131 | 319 | 0.305 | 16 | 85 | 198 | 0.298 | G | 1.126 (0.699-1.812) | 0.626 (1) |
| CAS |  | 75 | 265 | 519 | 0.401 | 45 | 124 | 306 | 0.351 | G | 1.272 (0.987-1.639) | 0.063 (1) |
| CAH |  | 48 | 205 | 494 | 0.307 | 26 | 132 | 283 | 0.332 | G | 0.873 (0.665-1.144) | 0.324 (1) |
| MDE |  | 40 | 95 | 230 | 0.483 | 49 | 83 | 196 | 0.425 | G | 0.88 (0.6-1.292) | 0.515 (1) |
| EUR | chr22:19963748:G:A  (rs4680; p.Val158Met) | 3145 | 6356 | 13034 | 0.500 | 1331 | 2502 | 5191 | 0.513 | A | 0.932 (0.878-0.989) | 0.020 (1) |
| AFR |  | 71 | 320 | 929 | 0.249 | 117 | 653 | 1617 | 0.275 | A | 1.000 (0.776-1.290) | 1.000 (1) |
| EAS |  | 147 | 796 | 2547 | 0.248 | 131 | 787 | 2345 | 0.262 | A | 0.973 (0.832-1.137) | 0.727 (1) |
| AAC |  | 19 | 142 | 279 | 0.323 | 83 | 347 | 793 | 0.323 | A | 1.019 (0.782-1.326) | 0.891 (1) |
| AJ |  | 249 | 625 | 1234 | 0.455 | 76 | 196 | 391 | 0.445 | A | 0.982 (0.79-1.221) | 0.872 (1) |
| AMR |  | 71 | 217 | 427 | 0.420 | 23 | 79 | 150 | 0.419 | A | 1.052 (0.738-1.5) | 0.778 (1) |
| SAS |  | 63 | 140 | 319 | 0.420 | 37 | 98 | 198 | 0.434 | A | 1.293 (0.863-1.938) | 0.213 (1) |
| CAH |  | 79 | 243 | 494 | 0.407 | 38 | 133 | 283 | 0.369 | A | 1.153 (0.888-1.496) | 0.286 (1) |
| CAS |  | 72 | 248 | 519 | 0.378 | 76 | 135 | 306 | 0.472 | A | 0.69 (0.539-0.883) | 0.003 (0.391) |
| MDE |  | 57 | 107 | 230 | 0.485 | 40 | 86 | 196 | 0.476 | A | 1.165 (0.794-1.71) | 0.434 (1) |
| EUR | chr22:19964281:G:A  (rs769224; p.Pro199=) | 5 | 516 | 13034 | 0.020 | 7 | 231 | 5191 | 0.024 | A | 0.854 (0.704-1.036) | 0.108 (1) |
| AFR |  | 8 | 142 | 929 | 0.085 | 17 | 274 | 1617 | 0.095 | A | 0.855 (0.547-1.336) | 0.492 (1) |
| EAS |  | 15 | 316 | 2547 | 0.069 | 7 | 223 | 2345 | 0.051 | A | 0.828 (0.63-1.09) | 0.179 (1) |
| AAC |  | 1 | 35 | 279 | 0.066 | 7 | 131 | 793 | 0.091 | A | 0.862 (0.537-1.382) | 0.537 (1) |
| AJ |  | 0 | 47 | 1234 | 0.019 | 0 | 13 | 391 | 0.017 | A | 0.869 (0.396-1.905) | 0.726 (1) |
| AMR |  | 1 | 23 | 427 | 0.029 | 0 | 8 | 150 | 0.027 | A | 3.19 (0.724-14.059) | 0.125 (1) |
| CAH |  | 1 | 41 | 494 | 0.044 | 1 | 15 | 283 | 0.030 | A | 1.161 (0.599-2.249) | 0.659 (1) |
| SAS |  | 1 | 11 | 319 | 0.020 | 0 | 4 | 198 | 0.010 | A | 1.754 (0.403-7.642) | 0.454 (1) |
| CAS |  | 1 | 33 | 519 | 0.034 | 0 | 25 | 306 | 0.041 | A | 0.665 (0.358-1.235) | 0.197 (1) |
| MDE |  | 0 | 14 | 230 | 0.030 | 0 | 13 | 196 | 0.033 | A | 0.912 (0.289-2.874) | 0.874 (1) |
| EUR | chr22:19964293:C:T  (rs165631; p.Leu203=) | 3 | 342 | 13034 | 0.013 | 1 | 125 | 5191 | 0.012 | T | 1.018 (0.792-1.309) | 0.886 (1) |
| AJ |  | 0 | 45 | 1234 | 0.018 | 0 | 16 | 391 | 0.020 | T | 1.661 (0.639-4.314) | 0.298 (1) |
| AMR |  | 0 | 15 | 427 | 0.018 | 0 | 4 | 150 | 0.013 | T | 0.826 (0.236-2.89) | 0.765 (1) |
| SAS |  | 0 | 12 | 319 | 0.019 | 0 | 6 | 198 | 0.015 | T | 0.85 (0.196-3.679) | 0.828 (1) |
| CAS |  | 0 | 17 | 519 | 0.016 | 0 | 14 | 306 | 0.023 | T | 0.613 (0.248-1.511) | 0.287 (1) |
| MDE |  | 1 | 20 | 230 | 0.048 | 0 | 14 | 196 | 0.036 | T | 0.683 (0.229-2.036) | 0.493 (1) |
| EUR | chr22:19969258:G:A  (rs165599; c.*522G>A) | 6301 | 5523 | 13034 | 0.695 | 2550 | 2145 | 5191 | 0.698 | A | 0.998 (0.937-1.063) | 0.942 (1) |
| AFR |  | 54 | 354 | 929 | 0.249 | 104 | 617 | 1617 | 0.255 | A | 1.051 (0.802-1.377) | 0.718 (1) |
| EAS |  | 631 | 1248 | 2547 | 0.496 | 569 | 1146 | 2345 | 0.492 | A | 1.021 (0.897-1.163) | 0.752 (1) |
| AAC |  | 34 | 135 | 279 | 0.364 | 99 | 365 | 793 | 0.355 | A | 1.037 (0.803-1.340) | 0.778 (1) |
| AJ |  | 453 | 588 | 1234 | 0.395 | 145 | 189 | 391 | 0.387 | A | 1.034 (0.830-1.287) | 0.766 (1) |
| AMR |  | 123 | 204 | 427 | 0.473 | 51 | 77 | 150 | 0.437 | A | 1.375 (0.975-1.94) | 0.069 (1) |
| SAS |  | 92 | 170 | 319 | 0.555 | 70 | 90 | 198 | 0.580 | A | 1.131 (0.762-1.677) | 0.542 (1) |
| CAH |  | 144 | 245 | 494 | 0.539 | 63 | 149 | 283 | 0.486 | A | 1.198 (0.931-1.542) | 0.161 (1) |
| CAS |  | 195 | 245 | 519 | 0.612 | 121 | 139 | 306 | 0.623 | A | 1.007 (0.785-1.291) | 0.958 (1) |
| MDE |  | 74 | 112 | 230 | 0.565 | 63 | 103 | 196 | 0.584 | A | 0.874 (0.579-1.318) | 0.520 (1) |
| EUR | chr22:19969340:G:A  (rs36082074; c.*604G>A) | 9 | 706 | 13034 | 0.028 | 5 | 271 | 5191 | 0.027 | A | 0.933 (0.785-1.109) | 0.431 (1) |
| AJ |  | 6 | 120 | 1234 | 0.053 | 1 | 47 | 391 | 0.063 | A | 0.686 (0.452-1.042) | 0.077 (1) |
| AMR |  | 1 | 13 | 427 | 0.018 | 1 | 9 | 150 | 0.037 | A | 0.46 (0.164-1.291) | 0.14 (1) |
| SAS |  | 1 | 17 | 319 | 0.030 | 1 | 12 | 198 | 0.035 | A | 1.543 (0.547-4.351) | 0.412 (1) |
| CAH |  | 0 | 14 | 494 | 0.014 | 1 | 10 | 283 | 0.021 | A | 0.693 (0.281-1.705) | 0.424 (1) |
| CAS |  | 2 | 28 | 519 | 0.031 | 1 | 15 | 306 | 0.028 | A | 1.032 (0.429-2.481) | 0.944 (1) |
| MDE |  | 1 | 25 | 230 | 0.059 | 0 | 31 | 196 | 0.079 | A | 0.592 (0.275-1.277) | 0.182 (1) |
| EUR | chr22:19969362:T:C  (rs35478083; c.*626T>C) | 20 | 951 | 13034 | 0.038 | 10 | 391 | 5191 | 0.040 | C | 0.968 (0.833-1.126) | 0.677 (1) |
| AFR |  | 6 | 131 | 929 | 0.077 | 9 | 272 | 1617 | 0.090 | C | 1.049 (0.693-1.590) | 0.820 (1) |
| EAS |  | 15 | 331 | 2547 | 0.071 | 11 | 237 | 2345 | 0.055 | C | 0.892 (0.683-1.165) | 0.401 (1) |
| AAC |  | 0 | 39 | 279 | 0.070 | 2 | 127 | 793 | 0.083 | C | 0.624 (0.379-0.028) | 0.064 (1) |
| AJ |  | 0 | 60 | 1234 | 0.024 | 0 | 18 | 391 | 0.023 | C | 1.06 (0.509-2.207) | 0.876 (1) |
| AMR |  | 3 | 36 | 427 | 0.049 | 1 | 14 | 150 | 0.053 | C | 1.822 (0.752-4.414) | 0.184 (1) |
| SAS |  | 2 | 25 | 319 | 0.045 | 0 | 19 | 198 | 0.048 | C | 0.602 (0.239-1.515) | 0.281 (1) |
| CAH |  | 3 | 53 | 494 | 0.060 | 1 | 36 | 283 | 0.067 | C | 0.928 (0.565-1.522) | 0.767 (1) |
| CAS |  | 3 | 53 | 519 | 0.057 | 1 | 37 | 306 | 0.064 | C | 0.808 (0.499-1.307) | 0.385 (1) |
| MDE |  | 0 | 25 | 230 | 0.054 | 0 | 22 | 196 | 0.056 | C | 1.08 (0.461-2.529) | 0.859 (1) |
| EUR | chr22:19969500:C:T  (rs165728; c.*764C>T) | 40 | 1295 | 13034 | 0.053 | 19 | 481 | 5191 | 0.050 | C | 0.984 (0.862-1.124) | 0.817 (1) |
| AFR |  | 0 | 86 | 929 | 0.046 | 7 | 165 | 1617 | 0.056 | C | 0.642 (0.376-1.096) | 0.104 (1) |
| EAS |  | 358 | 1192 | 2547 | 0.384 | 362 | 1069 | 2345 | 0.397 | C | 0.952 (0.831-1.091) | 0.482 (1) |
| AAC |  | 3 | 42 | 279 | 0.086 | 4 | 101 | 793 | 0.036 | C | 0.873 (0.556-1.369) | 0.553 (1) |
| AJ |  | 1 | 93 | 1234 | 0.038 | 2 | 45 | 391 | 0.063 | C | 0.602 (0.373-0.971) | 0.038 (1) |
| AMR |  | 26 | 117 | 427 | 0.198 | 2 | 39 | 150 | 0.143 | C | 1.414 (0.883-2.264) | 0.149 (1) |
| SAS |  | 4 | 76 | 319 | 0.132 | 1 | 44 | 198 | 0.118 | C | 0.913 (0.498-1.674) | 0.769 (1) |
| CAH |  | 15 | 105 | 494 | 0.137 | 7 | 53 | 283 | 0.118 | C | 1.082 (0.752-1.556) | 0.671 (1) |
| CAS |  | 14 | 164 | 519 | 0.186 | 6 | 77 | 306 | 0.146 | C | 1.26 (0.909-1.745) | 0.165 (1) |
| MDE |  | 2 | 21 | 230 | 0.055 | 0 | 13 | 196 | 0.033 | C | 2.475 (1.03-5.949) | 0.043 (1) |
| AFR | chr22:19961219:C:G  (rs11569715; c.-71C>G) | 0 | 68 | 929 | 0.037 | 1 | 109 | 1617 | 0.034 | G | 1.126 (0.561-2.259) | 0.738 (1) |
| AAC |  | 0 | 7 | 279 | 0.013 | 0 | 41 | 793 | 0.026 | G | 0.439 (0.16-1.207) | 0.111 (1) |
| AFR | chr22:19963714:C:T  (rs8192488; p.Ala146=) | 12 | 152 | 929 | 0.096 | 10 | 288 | 1617 | 0.096 | T | 0.916 (0.610-1.375) | 0.672 (1) |
| AAC |  | 1 | 39 | 279 | 0.074 | 2 | 107 | 793 | 0.070 | T | 1.276 (0.801-2.004) | 0.311 (1) |
| CAH |  | 2 | 27 | 494 | 0.031 | 1 | 17 | 283 | 0.034 | T | 0.936 (0.473-1.85) | 0.848 (1) |
| AFR | chr22:19968894:G:A  (rs9332380; c.*158G>A) | 2 | 63 | 929 | 0.036 | 2 | 142 | 1617 | 0.045 | A | 0.882 (0.431-1.806) | 0.732 (1) |
| AAC |  | 0 | 17 | 279 | 0.030 | 0 | 59 | 793 | 0.037 | A | 0.723 (0.347-1.509) | 0.388 (1) |
| AFR | chr22:19969244:T:C  (rs112696498; c.*508T>C) | 6 | 113 | 929 | 0.067 | 5 | 198 | 1617 | 0.064 | C | 1.128 (0.722-1.764) | 0.597 (1) |
| AAC |  | 2 | 34 | 279 | 0.068 | 1 | 61 | 793 | 0.040 | C | 1.538 (0.905-2.612) | 0.111 (1) |
| CAH |  | 1 | 21 | 494 | 0.023 | 1 | 17 | 283 | 0.034 | C | 1.051 (0.529-2.087) | 0.886 (1) |
| AFR | chr22:19969444:C:T  (rs35481270; c.*708C>T) | 0 | 15 | 929 | 0.008 | 1 | 40 | 1617 | 0.013 | T | 1.368 (0.550-3.402) | 0.500 (1) |
| AFR | chr22:19969728:T:C  (rs60537793; c.*992T>C) | 6 | 111 | 929 | 0.066 | 5 | 197 | 1617 | 0.064 | C | 1.080 (0.685-1.701) | 0.742 (1) |
| AAC |  | 2 | 32 | 279 | 0.065 | 1 | 57 | 793 | 0.037 | C | 1.658 (0.968-2.838) | 0.065 (1) |
| CAH |  | 0 | 20 | 494 | 0.020 | 1 | 12 | 283 | 0.025 | C | 1.057 (0.489-2.285) | 0.888 (1) |
| EAS | chr22:19962740:G:T  (rs6267; p.Ala72Ser) | 1 | 118 | 2547 | 0.025 | 0 | 91 | 2345 | 0.021 | T | 0.643 (0.415-0.998) | 0.049 (1) |
| AMR |  | 0 | 30 | 427 | 0.035 | 0 | 3 | 150 | 0.010 | T | 2.863 (0.642-12.774) | 0.168 (1) |
| CAS |  | 0 | 44 | 519 | 0.042 | 1 | 22 | 306 | 0.039 | T | 0.901 (0.508-1.599) | 0.722 (1) |
| CAH |  | 1 | 15 | 494 | 0.017 | 0 | 5 | 283 | 0.009 | T | 0.658 (0.186-2.331) | 0.517 (1) |
| AJ | chr22:19962643:C:T  (rs74745580; c.-34C>T) | 1 | 80 | 1234 | 0.033 | 0 | 13 | 391 | 0.017 | T | 1.911 (0.903-4.046) | 0.09 (1) |
| AJ | chr22:19968753:C:G  (rs368314788; c.*17C>G) | 0 | 47 | 1234 | 0.019 | 0 | 10 | 391 | 0.013 | G | 1.134 (0.497-2.586) | 0.766 (1) |
| AJ | chr22:19968866:C:T  (rs749822167; c.*130C>T) | 0 | 47 | 1234 | 0.019 | 0 | 10 | 391 | 0.013 | T | 1.134 (0.497-2.586) | 0.766 (1) |
| AMR | chr22:19962362:C:T  (rs6268; c.-165C>T) | 3 | 28 | 427 | 0.041 | 0 | 16 | 150 | 0.054 | T | 0.717 (0.338-1.521) | 0.386 (1) |
| EAS |  | 0 | 53 | 2547 | 0.011 | 0 | 51 | 2345 | 0.012 | T | 1.021 (0.52-2.007) | 0.951 (1) |
| SAS | chr22:19969314:G:A  (rs551937723; c.*578G>A) | 0 | 9 | 319 | 0.014 | 0 | 6 | 198 | 0.015 | A | 0.518 (0.116-2.323) | 0.39 (1) |

**Table S5.** Gene burden analysis of *COMT* variants. *Abbreviations: N Var, number of variants; AAC: African Admixed; AFR: African; AJ: Ashkenazi Jews; AMR: American Admixed; CAH, Complex Admixture History; CAS: Central Asian; EAS: East Asian; EUR: European; MDE: Middle Eastern; SAS: South Asian.*

| **Cohort** | **Kernel** | **MAF 1%** | | **MAF 3%** | | **Coding** | | **Missense** | |
| --- | --- | --- | --- | --- | --- | --- | --- | --- | --- |
|  |  | N Var | *p* | N Var | *p* | N Var | *p* | N Var | *p* |
| GP2 AAC | Skat | 171 | 0.975 | 223 | 0.997 | 9 | 0.689 | 4 | 0.415 |
|  | SkatO | 171 | 0.231 | 223 | 0.169 | 9 | 0.791 | 4 | 0.477 |
| GP2 AFR | Skat | 232 | 0.505 | 281 | 0.894 | 14 | 0.894 | 8 | 0.957 |
|  | SkatO | 232 | 0.028 | 281 | 0.085 | 14 | 0.795 | 8 | 1 |
| GP2 AJ | Skat | 73 | 0.250 | 95 | 0.216 | 10 | 0.097 | 3 | 0.069 |
|  | SkatO | 73 | 0.421 | 95 | 0.358 | 10 | 0.169 | 3 | 0.036 |
| GP2 AMR | Skat | 48 | 0.538 | 82 | 0.812 | 9 | 0.324 | 2 | 0.159 |
|  | SkatO | 48 | 0.769 | 82 | 1 | 9 | 0.105 | 2 | 0.151 |
| GP2 CAH | Skat | 123 | 0.885 | 176 | 0.630 | 10 | 0.301 | 4 | 0.469 |
|  | SkatO | 123 | 0.741 | 176 | 0.785 | 10 | 0.475 | 4 | 0.385 |
| GP2 MDE | Skat | 37 | 0.419 | 59 | 0.675 | 8 | 0.562 | 2 | 0.270 |
|  | SkatO | 37 | 0.611 | 59 | 0.583 | 8 | 0.178 | 2 | 0.271 |
| GP2 CAS | Skat | 65 | 0.444 | 95 | 0.720 | 7 | 0.391 | 2 | 0.723 |
|  | SkatO | 65 | 0.684 | 95 | 0.875 | 7 | 0.239 | 2 | 0.722 |
| GP2 EAS | Skat | 199 | 0.555 | 230 | 0.478 | 19 | 0.740 | 8 | 0.604 |
|  | SkatO | 199 | 0.702 | 230 | 0.646 | 19 | 0.250 | 8 | 0.404 |
| GP2 EUR | Skat | 641 | 0.923 | 692 | 0.987 | 33 | 0.023 | 18 | 0.020 |
|  | SkatO | 641 | 0.607 | 692 | 1 | 33 | 0.022 | 18 | 0.019 |
| GP2 SAS | Skat | 59 | 0.172 | 80 | 0.272 | 9 | 0.484 | 4 | 0.170 |
|  | SkatO | 59 | 0.361 | 80 | 0.444 | 9 | 0.591 | 4 | 0.212 |
| AMP-PD | Skat | 256 | 0.317 | 287 | 0.231 | 12 | 0.064 | 4 | 0.912 |
|  | SkatO | 256 | 0.543 | 287 | 0.250 | 12 | 0.010 | 4 | 0.781 |

**Table S6.** *COMT* exonic variants associated with MoCA scores adjusted by age at baseline, sex, education level, and five principal components in GP2 European genotyping data (N=568)*.*

*Abbreviations: A1, Allele 1, effect allele; BONF, Bonferroni adjusted P-value.*

| **Variant** | **A1** | **BETA (SE)** | ***p***  **(BONF)** |
| --- | --- | --- | --- |
| chr22:19962712:C:T  (rs4633; p.His62=) | C | -0.330 (0.149) | 0.027 (1) |
| chr22:19963748:G:A  (rs4680; p.Val158Met) | G | -0.338 (0.150) | 0.024 (1) |
| chr22:19962429:A:G  (rs6269; c.-98A>G) | G | -0.033 (0.154) | 0.831 (1) |
| chr22:19963684:C:G  (rs4818; p.Leu136=) | G | -0.031 (0.154) | 0.843 (1) |
| chr22:19964281:G:A  (rs769224; p.Pro199=) | A | 0.775 (0.554) | 0.162 (1) |
| chr22:19964293:C:T  (rs165631; p.Leu203=) | T | 0.103 (0.555) | 0.853 (1) |
| chr22:19969258:G:A  (rs165599; c.*522G>A) | G | -0.277 (0.167) | 0.103 (1) |
| chr22:19969340:G:A  (rs36082074; c.*604G>A) | A | 0.519 (0.484) | 0.284 (1) |
| chr22:19969362:T:C  (rs35478083; c.*626T>C) | C | -0.007 (0.409) | 0.987 (1) |
| chr22:19969500:C:T  (rs165728; c.*764C>T) | C | -0.591 (0.354) | 0.095 (1) |

**Table S7.** *COMT* exonic variants associated with MOCA scores adjusted by age at baseline, sex, education level, and five principal components in AMP-PD WGS (N=1,341). *Abbreviations: A1: Allele 1, effect allele; BONF, Bonferroni adjusted P-value.*

| **Variant** | **A1** | **BETA (SE)** | ***p* (BONF)** |
| --- | --- | --- | --- |
| chr22:19962429:A:G  (rs6269; c.-98A>G) | G | -0.236 (0.132) | 0.074 (1) |
| chr22:19962712:C:T  (rs4633; p.His62=) | T | 0.183 (0.131) | 0.163 (1) |
| chr22:19963684:C:G  (rs4818; p.Leu136=) | G | -0.218 (0.132) | 0.099 (1) |
| chr22:19963748:G:A  (rs4680; p.Val158Met) | A | 0.161 (0.131) | 0.218 (1) |
| chr22:19964281:G:A  (rs769224; p.Pro199=) | A | 0.203 (0.5496) | 0.712 (1) |
| chr22:19964293:C:T  (rs165631; p.Leu203=) | T | 0.968 (0.680) | 0.155 (1) |
| chr22:19969030:C:G  (rs9332381; c.*294C>G) | G | 0.278 (0.391) | 0.477 (1) |
| chr22:19969258:G:A  (rs165599; c.*522G>A) | A | 0.151 (0.142) | 0.287 (1) |
| chr22:19969340:G:A  (rs36082074; c.*604G>A) | A | 0.201 (0.411) | 0.625 (1) |
| chr22:19969362:T:C  (rs35478083; c.*626T>C) | C | 0.238 (0.394) | 0.546 (1) |
| chr22:19969500:C:T  (rs165728; c.*764C>T) | T | -0.163 (0.300) | 0.586 (1) |

**Table S8.** *COMT* exonic variants associated with MDS UPDRS Parts III (N=1,268) and IV (N=359) total scores, adjusted by age at baseline, sex, education level, and five principal components in GP2 European genotyping data.

*Abbreviations: A1, Allele 1, effect allele; BONF, Bonferroni adjusted P-value.*

| **MDS-UPDRS Part** | **Variant** | **A1** | **BETA (SE)** | ***p* (BONF)** |
| --- | --- | --- | --- | --- |
| III | chr22:19962429:A:G  (rs6269; c.-98A>G) | G | 0.034 (0.470) | 0.941 (1) |
| IV |  |  | 0.079 (0.267) | 0.768 (1) |
| III | chr22:19962712:C:T  (rs4633; p.His62=) | C | 0.154 (0.461) | 0.738 (1) |
| IV |  |  | 0.125 (0.265) | 0.636 (1) |
| III | chr22:19963684:C:G  (rs4818; p.Leu136=) | G | 0.035 (0.471) | 0.941 (1) |
| IV |  |  | 0.144 (0.268) | 0.591 (1) |
| III | chr22:19963748:G:A  (rs4680; p.Val158Met) | G | 0.152 (0.462) | 0.742 (1) |
| IV |  |  | 0.139 (0.264) | 0.599 (1) |
| III | chr22:19964281:G:A  (rs769224; p.Pro199=) | A | - 0.440 (1.776) | 0.804 (1) |
| IV |  |  | 0.044 (1.02) | 0.966 (1) |
| III | chr22:19964293:C:T  (rs165631; p.Leu203=) | T | 1.05 (1.901) | 0.581 (1) |
| IV |  |  | 1.294 (1.233) | 0.295 (1) |
| III | chr22:19969258:G:A  (rs165599; c.*522G>A) | G | -0.514 (0.518) | 0.321 (1) |
| IV |  |  | 0.020 (0.289) | 0.946 (1) |
| III | chr22:19969340:G:A  (rs36082074; c.*604G>A) | A | -2.335 (1.30) | 0.073 (1) |
| IV |  |  | 0.532 (0.707) | 0.452 (1) |
| III | chr22:19969362:T:C  (rs35478083; c.*626T>C) | C | -0.258 (1.244) | 0.835 (1) |
| IV |  |  | -0.239 (0.669) | 0.722 (1) |
| III | chr22:19969500:C:T  (rs165728; c.*764C>T) | C | -0.769 (1.058) | 0.467 (1) |
| IV |  |  | 0.570 (0.544) | 0.295 (1) |

**Table S9.** *COMT* exonic variants associated with MDS UPDRS III (N=1,666) and IV (N=1,794) scores adjusted by age at baseline, sex, education level and five principal components in AMP-PD WGS.

*Abbreviations: A1: Allele 1, effect allele; BONF, Bonferroni adjusted P-value.*

| **MDS-UPDRS Part** | **Variant** | **A1** | **BETA (SE)** | ***p* (BONF)** |
| --- | --- | --- | --- | --- |
| III | chr22:19962429:A:G  (rs6269; c.-98A>G) | G | 0.0112 (0.395) | 0.977 (1) |
| IV |  |  | -0.158 (0.140) | 0.258 (1) |
| III | chr22:19962712:C:T  (rs4633; p.His62=) | T | -0.139 (0.388) | 0.720 (1) |
| IV |  |  | 0.307 (0.138) | 0.026 (1) |
| III | chr22:19963684:C:G  (rs4818; p.Leu136=) | G | -0.053 (0.395) | 0.894 (1) |
| IV |  |  | -0.123 (0.140) | 0.377 (1) |
| III | chr22:19963748:G:A  (rs4680; p.Val158Met) | A | -0.038 (0.388) | 0.921 (1) |
| IV |  |  | 0.298 (0.138) | 0.031 (1) |
| III | chr22:19964281:G:A  (rs769224; p.Pro199=) | A | -1.065 (1.656) | 0.520 (1) |
| IV |  |  | 0.028 (0.584) | 0.962 (1) |
| III | chr22:19964293:C:T  (rs165631; p.Leu203=) | T | 1.872 (1.938) | 0.334 (1) |
| IV |  |  | -0.148 (0.731) | 0.839 (1) |
| III | chr22:19969030:C:G  (rs9332381; c.*294C>G) | G | -2.037 (1.115) | 0.068 (1) |
| IV |  |  | 0.230 (0.389) | 0.555 (1) |
| III | chr22:19969258:G:A  (rs165599; c.*522G>A) | A | -0.327 (0.432) | 0.450 (1) |
| IV |  |  | 0.170 (0.150) | 0.257 (1) |
| III | chr22:19969362:T:C  (rs35478083; c.*626T>C) | C | -2.056 (1.124) | 0.068 (1) |
| IV |  |  | 0.196 (0.392) | 0.617 (1) |
| III | chr22:19969340:G:A  (rs36082074; c.*604G>A) | A | -1.803 (1.135) | 0.112 (1) |
| IV |  |  | -0.579 (0.416) | 0.164 (1) |
| III | chr22:19969500:C:T  (rs165728; c.*764C>T) | T | -0.199 (0.899) | 0.824 (1) |
| IV |  |  | 0.666 (0.320) | 0.038 (1) |

**Figure S1.** Locus zoom plot for *COMT* variants versus PD risk. **(A)** Data was obtained from the latest European PD GWAS meta-analysis excluding 23andMe data, consisting of 15,056 PD cases, 18,618 UK Biobank proxy-cases, and 449,056 healthy controls [(1)](https://www.zotero.org/google-docs/?fxeUOA). **(B)** Data was obtained from the East Asian PD GWAS, consisting of 6,724 PD cases and 24,851 healthy controls [(2)](https://www.zotero.org/google-docs/?5cJPRb). **(C)** Data was obtained from the Latin American PD GWAS, consisting of 807 PD cases and 690 healthy controls [(3)](https://www.zotero.org/google-docs/?9V4ECi). **(D)** Data was obtained from the African PD GWAS excluding 23andMe data, consisting of 1,200 PD cases and 2,445 healthy controls [(4)](https://www.zotero.org/google-docs/?EbkTQt). **(E)** Data was obtained from the multi-ancestry PD GWAS excluding 23andMe data, consisting of 25,374 PD cases, 18,618 proxy-cases, and 571,138 healthy controls [(5)](https://www.zotero.org/google-docs/?ypPplj). p-values on the log-10 scale are plotted along the horizontal axis with the gene names and size of the flanking region. The most strongly associated SNPs are indicated by a purple diamond and pairwise LD (r2) with these SNPs are indicated by dotted color as described in the legend in the upper right corner. The right vertical axis indicates the regional recombination rate (cM/Mb) which is overlaid in blue. Abbreviations: LD, linkage disequilibrium; PD, Parkinson’s disease; SNP, single nucleotide polymorphism.


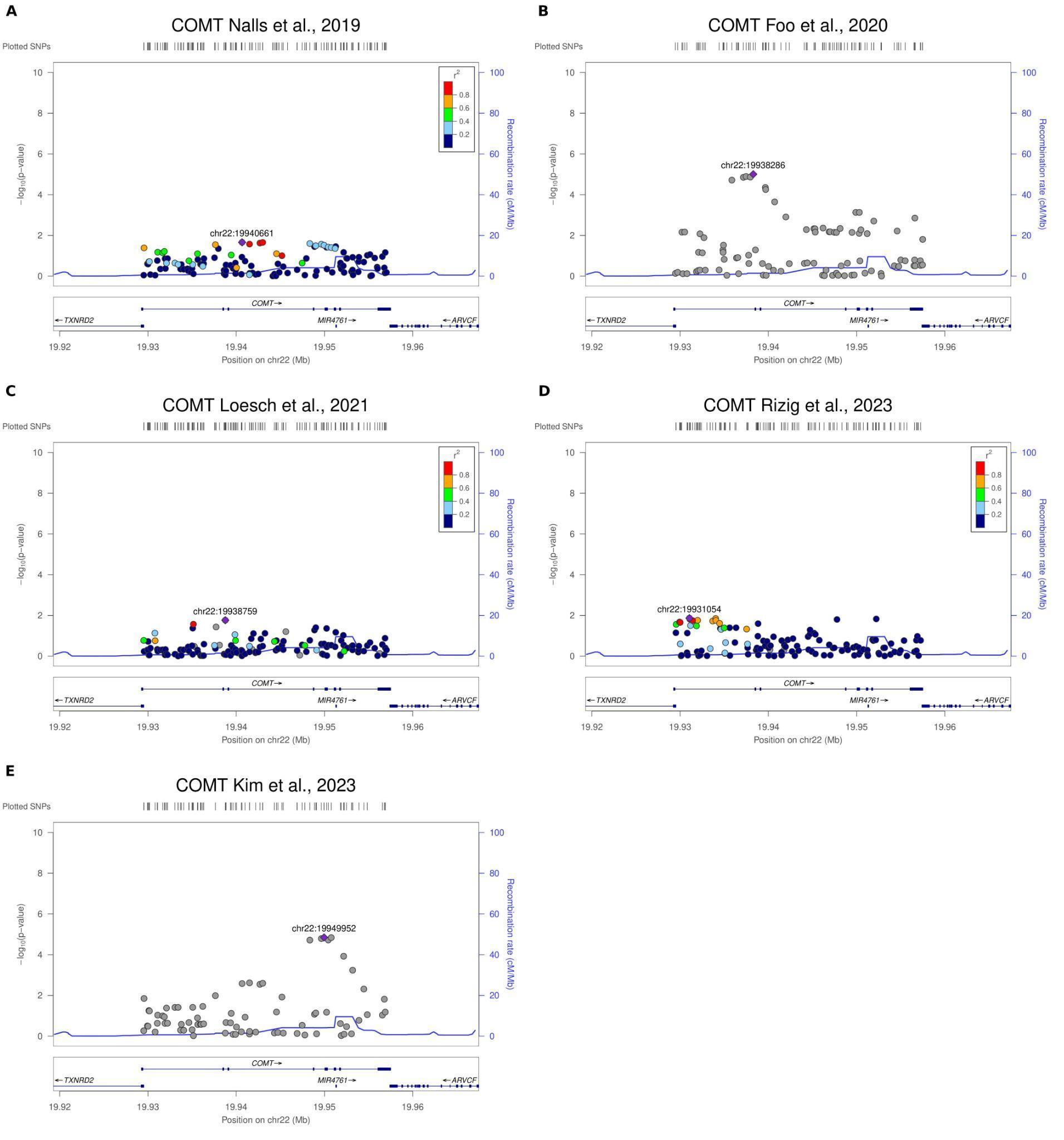


**Figure S2.** Levodopa-induced Dyskinesias (LID) survival analysis among 463 PD patients from GP2 European genotyping data.

**
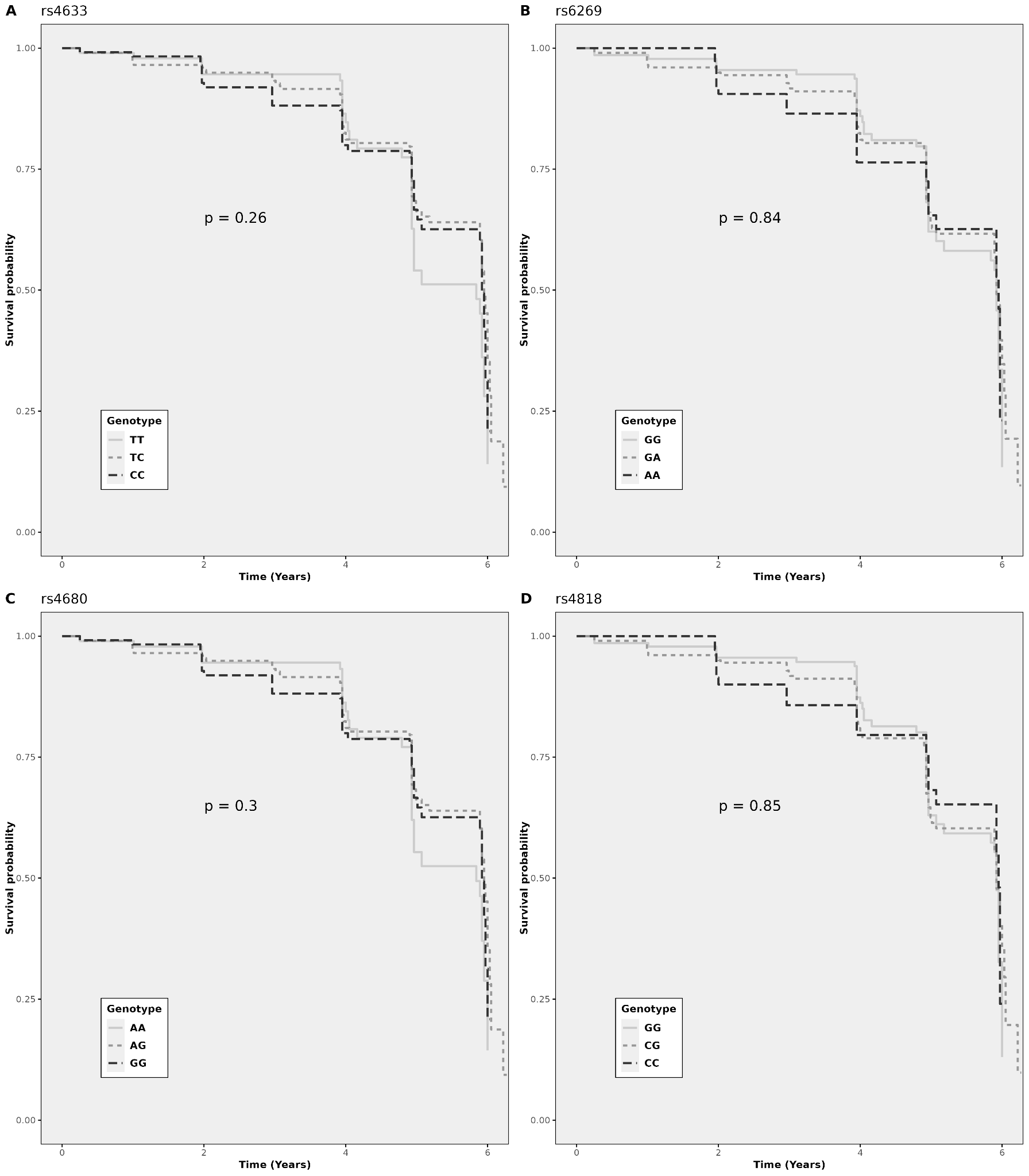
**

**Supplementary References**

1. Vitale D, Koretsky MJ, Kuznetsov N, Hong S, Martin J, James M, et al. GenoTools: An Open-Source Python Package for Efficient Genotype Data Quality Control and Analysis. G3 (Bethesda) [Internet]. 2024; Available from: http://dx.doi.org/10.1093/g3journal/jkae268

2. Wang K, Li M, Hakonarson H. ANNOVAR: functional annotation of genetic variants from high-throughput sequencing data. Nucleic Acids Res. 2010;38:e164.

3. Zhan X, Hu Y, Li B, Abecasis GR, Liu DJ. RVTESTS: an efficient and comprehensive tool for rare variant association analysis using sequence data. Bioinformatics. 2016;32:1423–6.

4. Hill A, Loh P-R, Bharadwaj RB, Pons P, Shang J, Guinan E, et al. Stepwise Distributed Open Innovation Contests for Software Development: Acceleration of Genome-Wide Association Analysis. Gigascience. 2017;6:1–10.
